# Supplementary material for: Multivariate Curve Resolution Alternating Least Squares Analysis of In Vivo Skin Raman Spectra
Source: Sensors (Basel). 2022 Dec 7;22(24):9588. doi: 10.3390/s22249588 (PMC9785721; doi:10.3390/s22249588)
Supplement: Supplementary file 1 [file sensors-22-09588-s001.zip › sensors-1965599-SI.pdf]

SUPPLEMENTARY SECTION

This Supplementary Section presents spectral profiles obtained by MCR analysis of pre-processed in vivo Raman spectra of normal skin and diseases. As discussed in Section 2.2, the spectra of normal skin were divided into a different number of components from 4 to 30, and the spectra of diseases – into 5 components. The result of the MCR analysis of normal skin Raman spectra is shown in Supplementary Figures S1, S2, and the result of the analysis of disease Raman spectra – in Supplementary Figure S3. The number of components to be unmixed is indicated by a number, for example “MCR into 4 components” means that Raman spectra are unmixed into four components. The diseases are indicated by letters on the diagrams.

This Supplementary Section also presents concentrations of the components obtained by MCR analysis of in vivo Raman spectra of normal skin and diseases using a basis (see Section 3.3). The results are presented using box-plot diagrams (see Supplementary Figure S4). The groups of spectra are indicated by letters on the diagrams.

It is important to understand that the values of the concentrations and intensities of the spectra were obtained in arbitrary units and vary for each individual analysis. The reason for this is the ambiguity of the bilinear transformation, as well as the fact that the raw Raman spectra are also recorded in arbitrary units. Thus, the Raman spectra were normalized with rescaling (min-max normalization, when the lowest intensity is set to 0, and the highest is set to 1). And the concentrations were recalculated so that the product of the normalized spectral profile and the concentration remained the same.

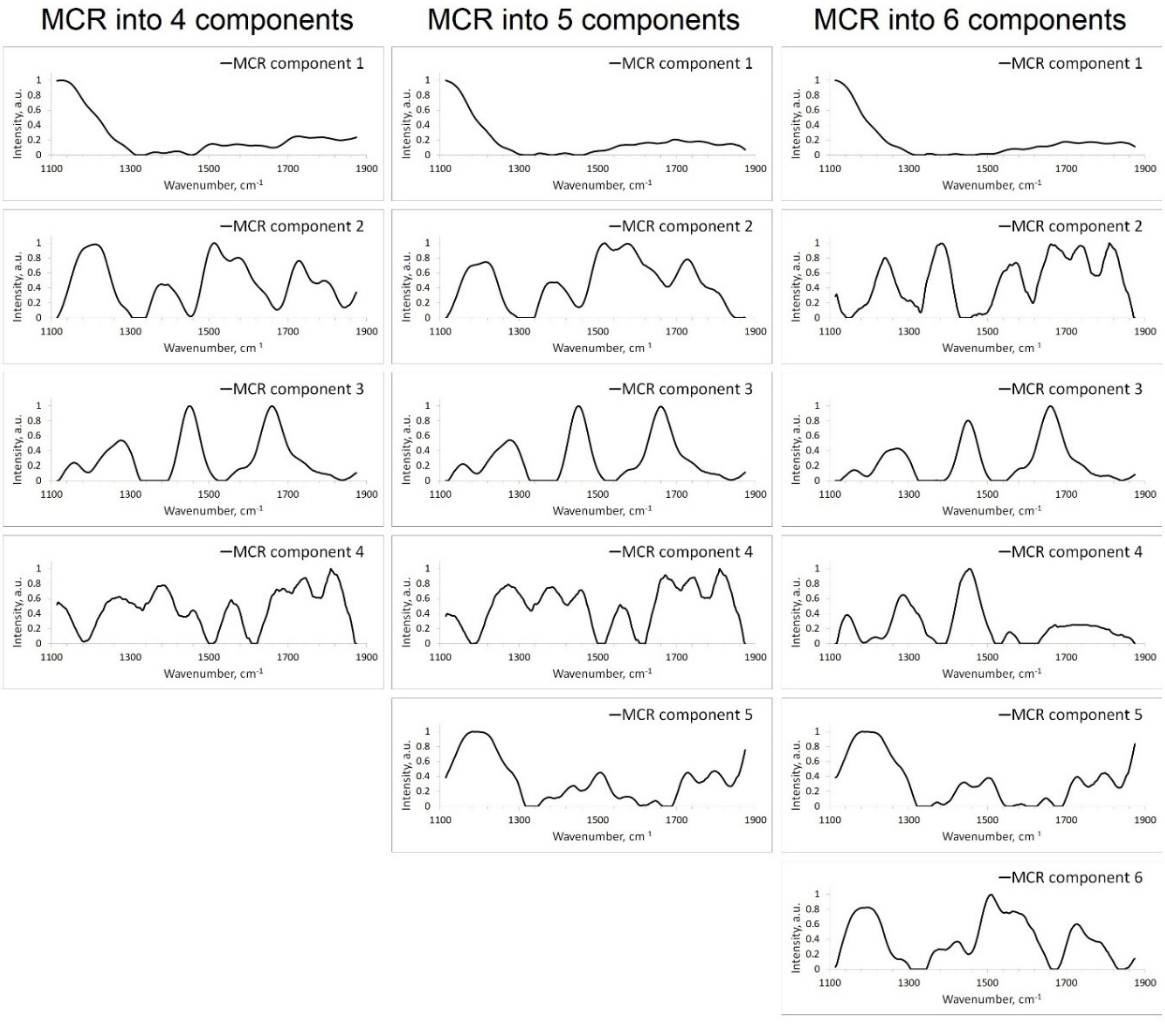

Supplementary Figure S1. The result of MCR analysis of normal skin Raman spectra.

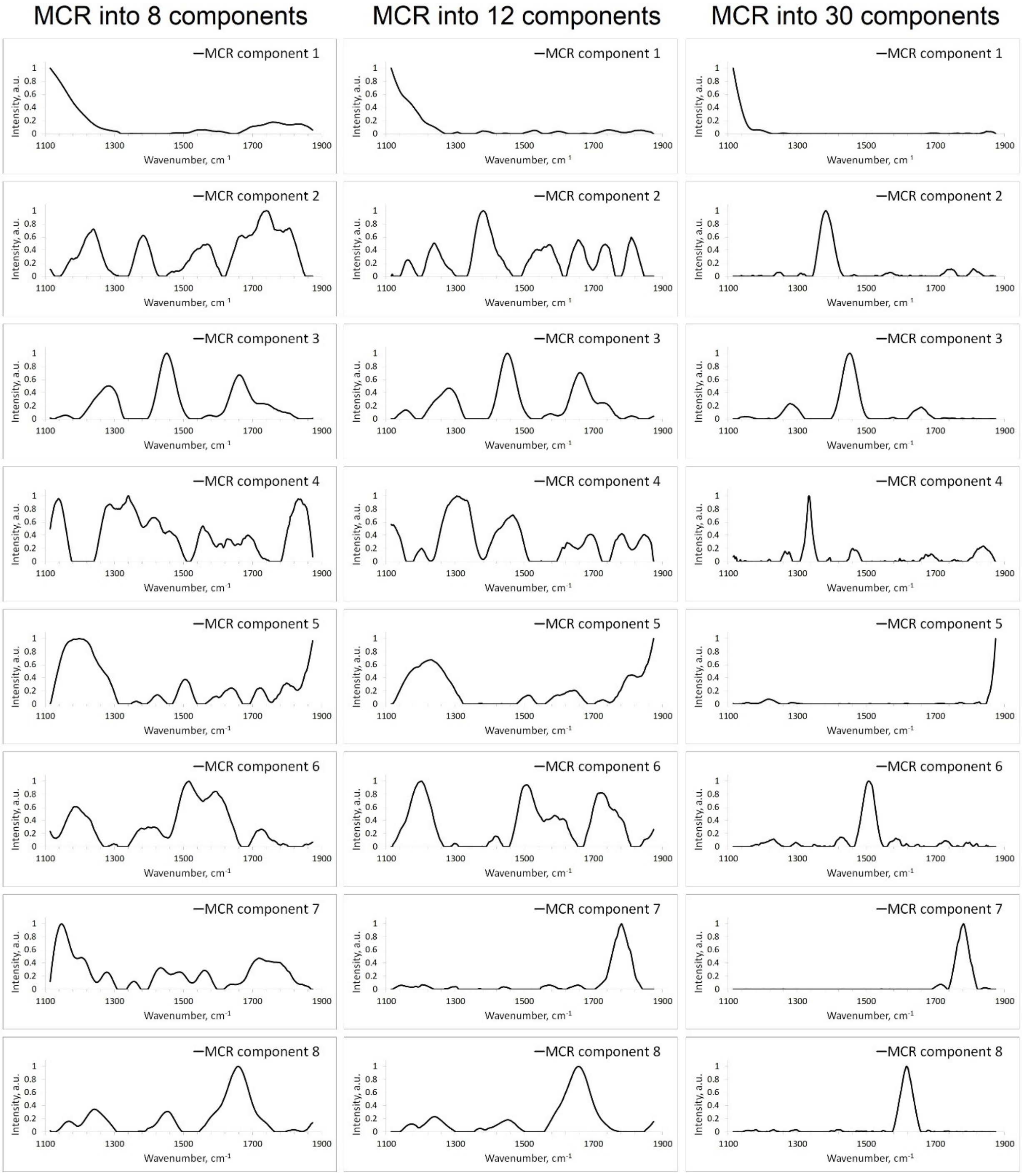

Supplementary Figure S2. The result of MCR analysis of normal skin Raman spectra.

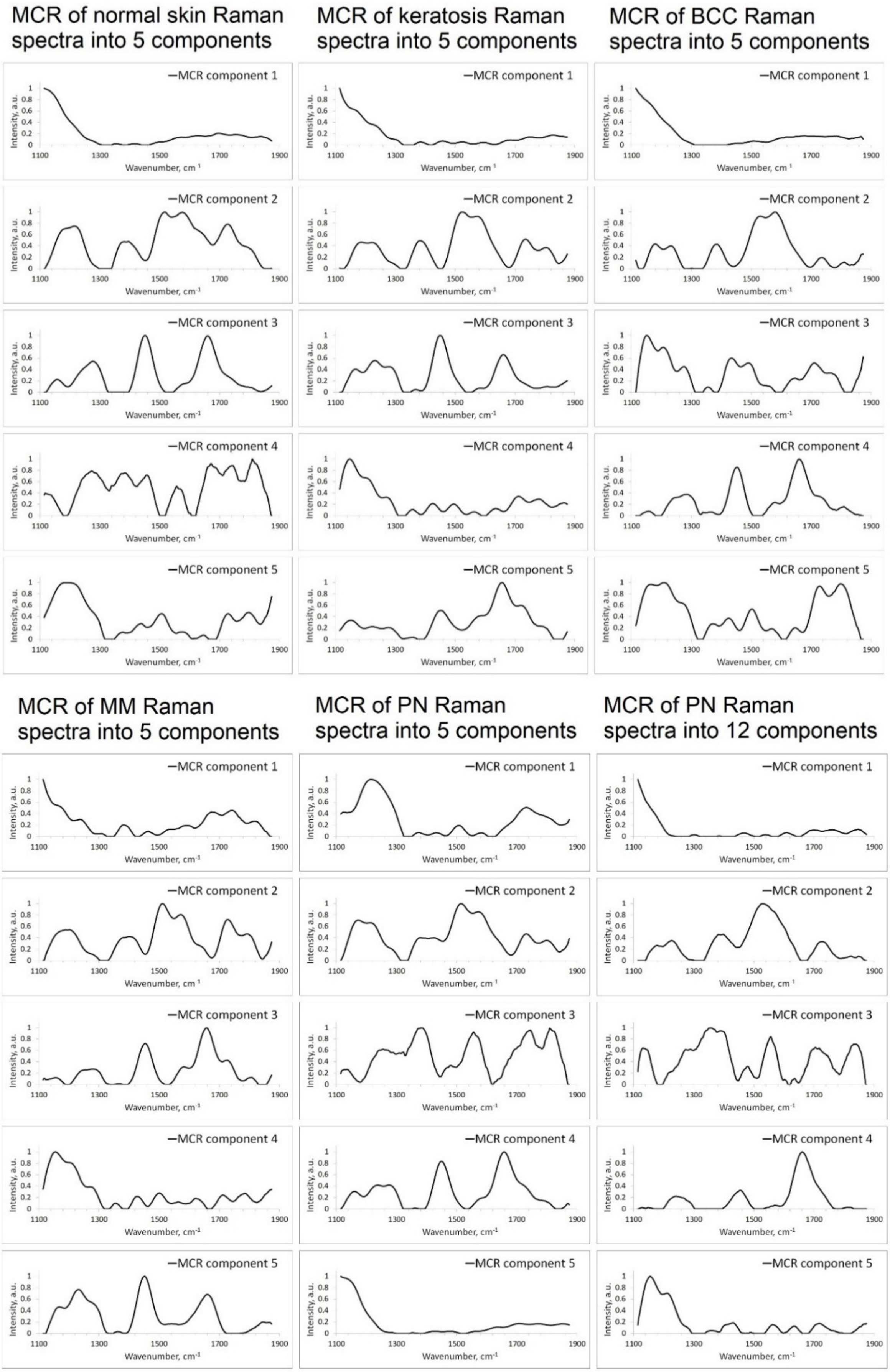

Supplementary Figure S3. The result of MCR analysis of disease Raman spectra (BCC is basal cell carcinoma, MM is malignant melanoma, PN is pigmented nevus).

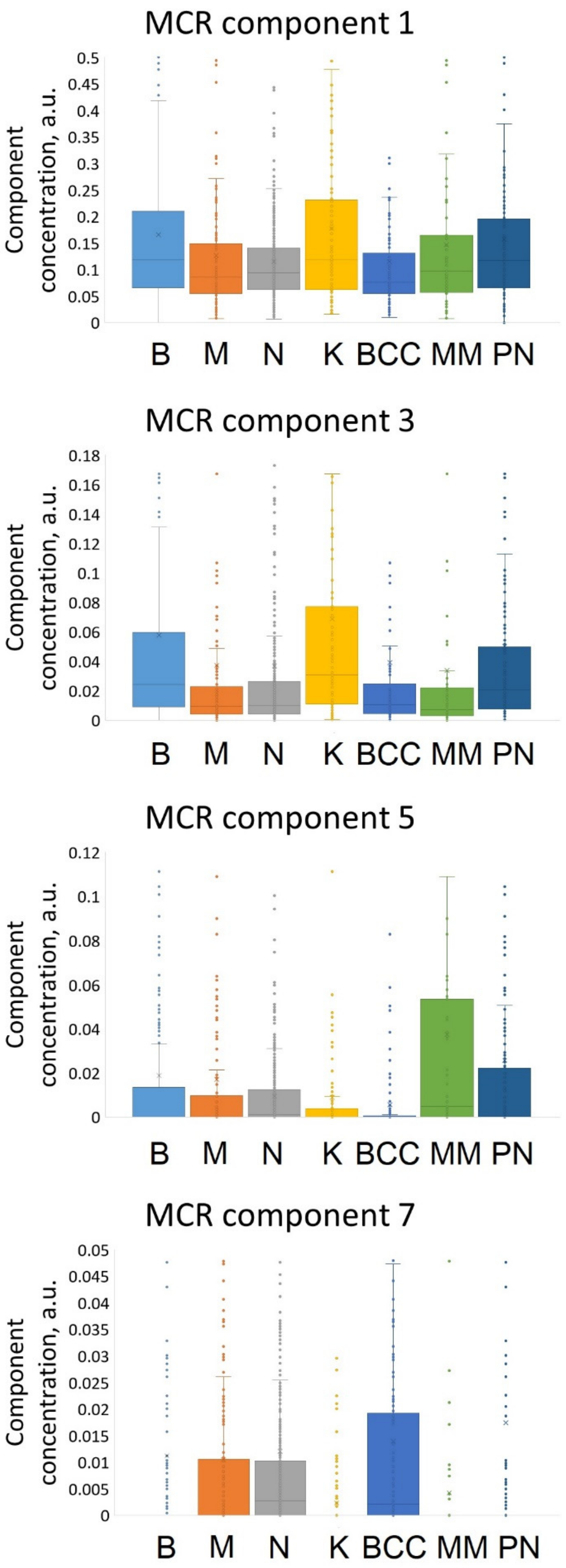

Supplementary Figure S4. Box-plot diagrams of skin component concentrations obtained by MCR analysis using a basis (B is benign neoplasms, M is malignant neoplasms, N is normal skin, BCC is basal cell carcinoma, MM is malignant melanoma, PN is pigmented nevus).
